# Supplementary material for: Characterization of Extended-Spectrum Beta-Lactamase-Producing Escherichia coli Isolates from Jurong Lake, Singapore with Whole-Genome-Sequencing
Source: Int J Environ Res Public Health. 2021 Jan 22;18(3):937. doi: 10.3390/ijerph18030937 (PMC7908357; doi:10.3390/ijerph18030937)
Supplement: Supplementary file 1 [file ijerph-18-00937-s001.pdf]

Characterization of Extended-Spectrum Beta-Lactamase-Producing *Escherichia coli* isolates from Jurong Lake, Singapore with Whole-Genome-Sequencing

Yang Zhong[1,2], Siyao Guo[1,2], Kelyn Lee Ghee Seow[1,2], Glendon Ong Hong Ming[1,2], Joergen Schlundt[1,2*]

[1] Nanyang Technological University Food Technology Centre (NAFTEC), 62 Nanyang Drive Singapore 637459

[2] School of Chemical and Biomedical Engineering, Nanyang Technological University, 62 Nanyang Drive Singapore 637459

*Corresponding author

Strain ID	GC content /%	Genome size /bp	Kmer result	MLST	Plasmid Ori detected
**J1E1**	50.6	5,111,241	*E. coli*	68	IncFII(pCoo)*, Col156*
**J1E2**	50.5	4,953,866	*E. coli*	457	Incl1*,ColpVC*
**J1E3**	50.3	5,176,493	*E. coli*	127	IncFII(pRSB107), IncFIB*,IncFIA,Col156*
**J1E4**	50.5	4,866,444	*E. coli*	10	IncFIA(HI1),IncHl1A,IncHl1B(R27)
**J1G1**	50.3	5,408,697	*E. coli*	648	IncFII(pRSB107), IncFIB*,IncFIA*,Col156*,Col(MG828)*
**J2E1**	50.7	5,006,788	*E. coli*	131	IncFII(pRSB107), IncFIB*,IncFIA,Col156*
**J2E2**	50.6	5,088,348	*E. coli*	38	IncFII(pHN7A8)*,ColpVC*
**J2E3**	50.5	5,248,876	*E. coli*	1730	IncFIB*,IncFII,IncHl2,IncHl2A*
**J2E4**	50.6	4,886,227	*E. coli*	215	IncFIA(HI1)*,IncHl1*,IncR*, IncX4, IncFIA*

Table 1: Basic Information of the isolates. *: without 100% identify.

Isolates ID	Aminoglycoside	Beta-lactam	Colistin	Fluoroquinolone	Fosfomycin	MLS
J1E1		$bla_{\text{CTX-M-15}}$			*fosA4*	*mdf(A)**,*mph(A)*
J1E2		$bla_{\text{CTX-M-8}}$				*mdf(A)**
J1E3	*aph(3'')-lb, aadA5, aac(3)-lld*, aph(6)-ld*	$bla_{\text{TEM-1B}}$,$bla_{\text{CTX-M-15}}$		*gyrA* p.S83L		*mdf(A)**,*mph(A)*
J1E4	*aadA1**	$bla_{\text{TEM-1B}}$,$bla_{\text{CTX-M-15}}$	*mcr*-1.1	*qnrS1, gyrA* p.S83L		*mdf(A)**
J1G1		$bla_{\text{CTX-M-14}}$		*gyrA* p.S83L, p.D87N, *parE* p.S458A, *parC* p.S80I		*mdf(A)**
J2E1	*aph(3'')-lb, aadA5, aph(6)-ld*	$bla_{\text{CTX-M-27}}$		*parC* p.S80I,*parE* p.E460D, *gyrA* p.D87N, *gyrA* p.S83L		*mdf(A)**,*mph(A)*
J2E2		$bla_{\text{CTX-M-15}}$		*qnrS1*		*mdf(A)**
J2E3	*aph(3')-la*, aadA2, aac(3)-lld*, aph(6)-ld,aph(3'')-lb,aadA1**	$bla_{\text{CTX-M-55}}$	*mcr*-3.1	*qnrS1**		*mdf(A)**,*mph(A)*
J2E4		$bla_{\text{CTX-M-15}}$		*qnrS1*		*mdf(A)**

Isolates ID	Phenicol	Sulphonamide	Tetracycline	Trimethoprim	Total resistant genes number
J1E1					4
J1E2					2
J1E3		*sul1,sul2*	*tet(A)*	*dfrA17*	12
J1E4	*floR**	*sul3*	*tet(A)*		9
J1G1				*dfrA17*	3
J2E1		*sul1,sul2*	*tet(A)*	*dfrA17*	10
J2E2					3
J2E3	*floR*,catA2**	*sul1,sul3*	*tet(A)**	*dfrA12*	17
J2E4					3

Table 2: Acquired resistance genes and resistance-related mutations detected in isolates by ResFinder. MLS: macrolide, lincosamide, and streptogramin B,*: identification below 100%.

Isolates ID	Aminoglycoside		Beta-lactam		Colistin		Fluoroquinolone	
	Genotype	Phenotype (Gentamic, MIC)	Genotype	Phenotype (MIC)	Genotype	Phenotype (MIC)	Genotype	Phenotype (Ciprofloxacin, MIC)
J1E1	-	S	+	R	-	S	-	S
J1E2	-	S	+	R	-	S	-	S
J1E3	+	R	+	R	-	S	+	S
J1E4	+	S	+	R	+	R	+	R
J1G1	-	S	+	R	-	S	+	R
J2E1	+	S	+	R	-	S	+	R
J2E2	-	S	+	R	-	S	+	S
J2E3	+	S	+	R	+	S	+	S
J2E4	-	S	+	R	-	S	+	R

Isolates ID	Phenicol		Sulphonamide		Tetracycline	
	Genotype	Phenotype (Chloramphenicol, Disc diffusion)	Genotype	Phenotype (Trimethoprim-sulfamethoxazole, Disc Diffusion)	Genotype	Phenotype (Disc Diffusion)
J1E1	-	S	-	R	-	S
J1E2	-	S	-	S	-	S
J1E3	-	S	+	R	+	R
J1E4	+	R	+	S	+	R
J1G1	-	S	-	S	-	S
J2E1	-	S	+	R	+	R
J2E2	-	S	-	S	-	S
J2E3	+	R	+	R	+	R
J2E4	-	S	-	S	-	S

Table 3. the comparison of phenotypic and genotypic resistance. +/-: with/without resistance genes or related mutations, S: sensitive, R: resistance, Grey highlight: disagreement between genotype and phenotype.

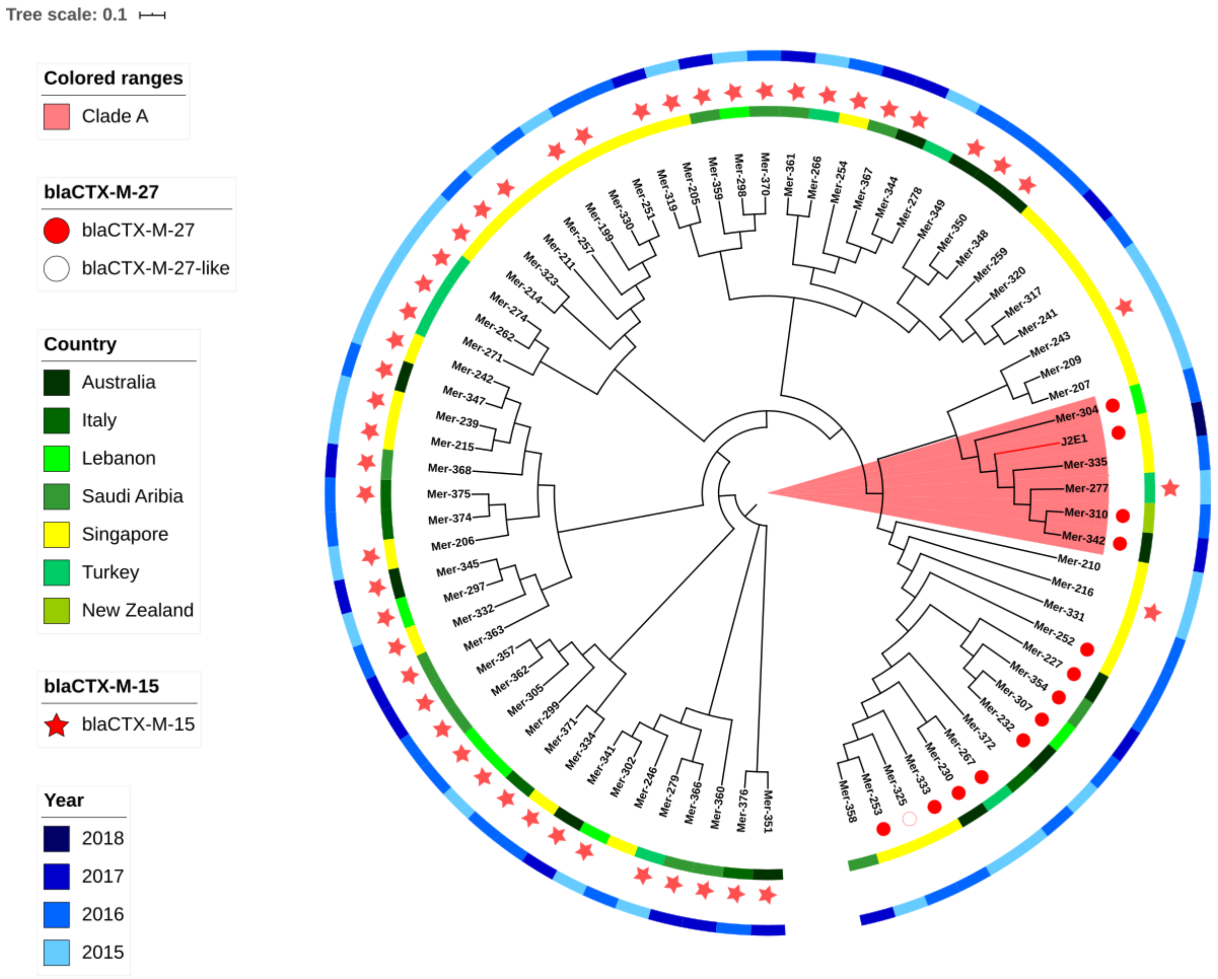

Fig 1. the phylogenetic tree of ST131 isolates from both the clinic and reservoir based on core genome SNPs. The tree was built with E. coli MG1655 as the reference genome and branch length was ignored. J2E1 was isolated in this research and or the other isolates were from NCBI. The metadata was shown in supplementary

Isolate ID	Contigs information		Resistance information		Insert sequence Information				
	Contigs ID	contigs length/bp	AMR genes	Position on the contigs	Insert sequence ID	Insert length/bp	Insert position	IS family	Acession
J1E1	27	269951	*bla*$_{CTX-M-15}$	197172..198047	ISEcp1	1656	195468..197123	IS1380	AJ242809
J1E3	151	128211	*bla*$_{CTX-M-15}$	86273..87148	ISEcp1	1656	84569..86224	IS1380	AJ242809
J1E4	28	128728	*bla*$_{CTX-M-15}$	43925..44800	ISEcp1	1656	42221..43876	IS1380	AJ242809
J1G1	80	110660	*bla*$_{CTX-M-14}$	83884..84759	ISEcp1	1656	82186..83841	IS1380	AJ242809
J2E2	31	289403	qnrS1	242382..243038	ISKpn19*	2851	238783..241633	ISKra4	NC_010886
			bla$_{CTX-M-15}$	247679..248554	ISEcp1*	1656	248603..250258	IS1380	AJ242809
J2E4	220	716717	qnrS1	555846..556502	ISEcp1	1656	562067..563722	IS1380	AJ242809
			bla$_{CTX-M-15}$	561143..562018					

Table 4. Insert sequence and their location detected by ISFinder. *: identification below 100%.

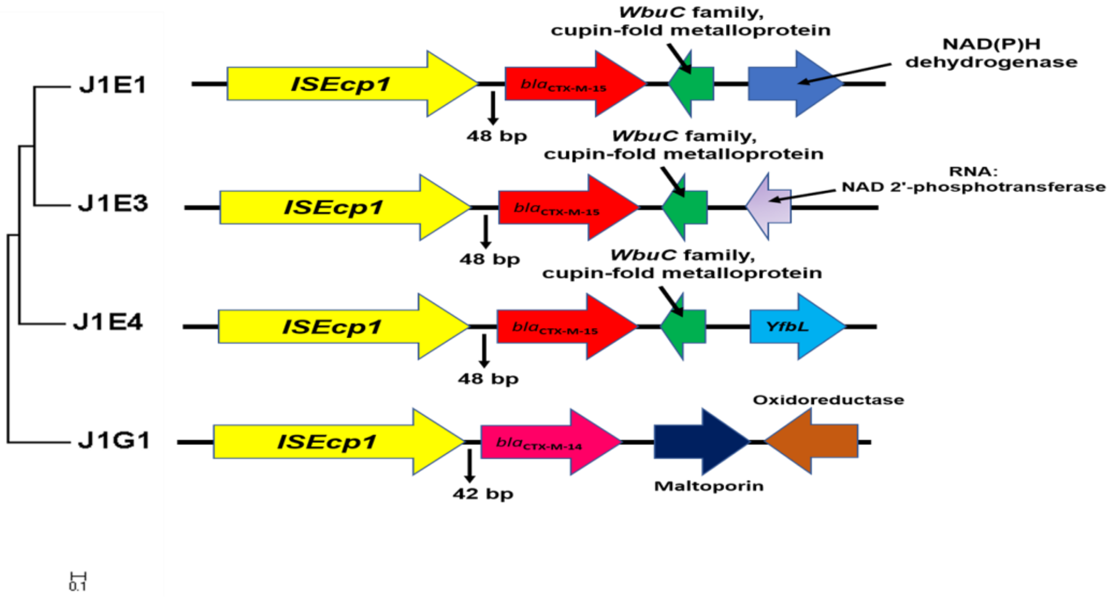

Fig 2. The genetic environment surrounding *bla*$_{CTX-M}$ genes located on the chromosome. The maximum likelihood tree was built based on the alignment of 5000 bp fragments contain insert sequence and *bla*$_{CTX-M}$ gene extract from the chromosome. The gene annotation and direction were generated by the RAST server and further corrected with NCBI blast. The conversed gene cluster contains *ISEcp1*, *bla*$_{CTX-M-15,}$ and *WubC* family metalloprotein.

Antimicrobials	J1E4 (Donor)	J53Coli (Transconjugates)	J53 (Recept)
**Ceftriaxone**	>128	>128	<1
**Meropenem**	<1	<1	<1
**Cephalothin**	>16	>16	>16
**Cefpodoxime**	>32	>32	2
**Ciprofloxacin**	2	>2	<1
**Cefotaxime**	>64	>64	<0.25
**Gentamicin**	<4	<4	<4
**Cefotaxime / clavulanic acid**	<0.12\4	<0.12\4	<0.12\4
**Ampicillin**	>16	>16	<8
**Ceftazidime**	>128	32	1
**Cefazolin**	>16	>16	<8
**Ceftazidime / clavulanic acid**	0.25\4	0.5\4	0.5\4
**Imipenem**	<0.5	1	1
**Piperacillin / tazobactam constant 4**	>64\4	<4\4	<4\4
**Cefepime**	>16	>16	<1
**Colistin**	4	8	<0.25
**Cefoxitin**	<4	8	<4

Table 5. The MIC comparison among J1E4, J53, and the transconjugants. The MIC was determined with the microdilution methods. The successful transconjugants gain multi-drug resistance from the donor strain J1E4 including the resistance of colistin, 3rd generation cephalosporins, and ciprofloxacin.

Supplementary Data:

Antimicrobials	J1E1	J1E2	J1E3	J1E4	J1G1	J2E1	J2E2	J2E3	J2E4	Breakpoint (CLSI-M100-ED30, Table 2A)
**Ceftriaxone**	>128	>128	>128	>128	>128	>128	>128	>128	>128	4
**Meropenem**	<1	<1	<1	<1	<1	<1	<1	<1	<1	4
**Cephalothin**	>16	>16	>16	>16	>16	>16	>16	>16	>16	N.A.
**Cefpodoxime**	>32	>32	>32	>32	>32	>32	>32	>32	>32	8
**Ciprofloxacin**	<1	<1	<1	2	>2	>2	<1	<1	>2	1
**Cefotaxime**	>64	>64	>64	>64	>64	>64	>64	>64	>64	4
**Gentamicin**	<4	<4	>16	<4	<4	<4	<4	<4	<4	16
**Cefotaxime / clavulanic acid**	32\4	<0.12\4	<0.12\4	<0.12\4	<0.12\4	<0.12\4	<0.12\4	<0.12\4	<0.12\4	N.A.
**Ampicillin**	>16	>16	>16	>16	>16	>16	>16	>16	>16	32
**Ceftazidime**	>128	1	32	>128	1	4	8	64	>128	16
**Cefazolin**	>16	>16	>16	>16	>16	>16	>16	>16	>16	32
**Ceftazidime / clavulanic acid**	4\4	<0.12\4	<0.12\4	0.25\4	0.25\4	<0.12\4	<0.12\4	0.25\4	8\4	N.A.
**Imipenem**	<0.5	<0.5	<0.5	<0.5	<0.5	<0.5	<0.5	<0.5	<0.5	4
**Piperacillin / tazobactam constant 4**	>64\4	<4\4	<4\4	>64\4	<4\4	<4\4	4\4	<4\4	>64\4	128\4
**Cefepime**	>16	>16	>16	>16	>16	>16	>16	>16	>16	16
**Cefoxitin**	16	8	<4	<4	16	8	8	16	<4	32

Table S1: MIC of selected antimicrobials. Based on the breakpoint of CLSI-M100, Red: resistant, yellow: intermedium, green: sensitive.

	Time	Country	Patient sex	Age	Diseases	Source	SRA number
Mer-376	2016	Italy	female	89	bacterial infection	blood	7828786
Mer-375	2016	Italy	female	84	bacterial infection	blood	7828661
Mer-374	2016	Italy	female	81	bacterial infection	blood	7828658
Mer-372	2016	Italy	male	62	bacterial infection	blood	7828660
Mer-371	2016	Italy	male	66	bacterial infection	blood	7828655
Mer-370	2016	Saudi Aribia	female	61	bacterial infection	blood	7828746
Mer-368	2017	Saudi Aribia	male	85	bacterial infection	blood	7828744
Mer-367	2017	Saudi Aribia	female	52	bacterial infection	blood	7828743
Mer-366	2017	Saudi Aribia	male	80	bacterial infection	blood	7828638
Mer-363	2017	Saudi Aribia	male	94	bacterial infection	blood	7828739
Mer-362	2016	Saudi Aribia	male	83	bacterial infection	blood	7828820
Mer-361	2017	Saudi Aribia	female	25	bacterial infection	blood	7828821
Mer-360	2017	Saudi Aribia	male	50	bacterial infection	blood	7828822
Mer-359	2017	Saudi Aribia	male	55	bacterial infection	blood	7828823
Mer-358	2017	Saudi Aribia	male	80	bacterial infection	blood	7828824
Mer-357	2017	Saudi Aribia	female	76	bacterial infection	blood	7828825
Mer-354	2017	Saudi Aribia	male	89	bacterial infection	blood	7828828
Mer-351	2017	Australia	female	77	bacterial infection	blood	7828742
Mer-350	2016	Australia	female	80	bacterial infection	blood	7828751
Mer-349	2016	Australia	female	72	bacterial infection	blood	7828752
Mer-348	2016	Australia	male	82	bacterial infection	blood	7828753
Mer-347	2016	Australia	female	63	bacterial infection	blood	7828754
Mer-345	2017	Australia	male	73	bacterial infection	blood	7828748
Mer-344	2017	Australia	female	78	bacterial infection	blood	7828749

Mer-342	2017	Australia	male	84	bacterial infection	blood	7828715
Mer-341	2017	Australia	female	78	bacterial infection	blood	7828714
Mer-335	2016	Singapore	male	78	bacterial infection	blood	7828724
Mer-334	2016	Singapore	female	38	bacterial infection	blood	7828723
Mer-333	2016	Singapore	male	81	bacterial infection	blood	7828722
Mer-332	2016	Singapore	male	76	bacterial infection	blood	7828721
Mer-331	2016	Singapore	female	59	bacterial infection	blood	7828736
Mer-330	2016	Singapore	female	39	bacterial infection	blood	7828737
Mer-325	2016	Singapore	male	56	bacterial infection	blood	7828730
Mer-323	2016	Singapore	female	78	bacterial infection	blood	7828728
Mer-320	2017	Singapore	male	85	bacterial infection	blood	7828708
Mer-319	2017	Singapore	female	90	bacterial infection	blood	7828707
Mer-317	2016	Singapore	female	40	bacterial infection	blood	7828703
Mer-310	2016	New Zealand	male	89	bacterial infection	blood	7828701
Mer-307	2016	Lebanon	male	87	bacterial infection	blood	7828677
Mer-305	2016	Lebanon	male	76	bacterial infection	blood	7828679
Mer-304	2016	Lebanon	male	52	bacterial infection	blood	7828680
Mer-302	2015	Lebanon	female	76	bacterial infection	blood	7828682
Mer-299	2015	Lebanon	male	75	bacterial infection	blood	7828645
Mer-298	2015	Lebanon	female	74	bacterial infection	blood	7828644
Mer-297	2015	Lebanon	male	54	bacterial infection	blood	7828654
Mer-279	2015	Turkey	male	30	bacterial infection	blood	7828838
Mer-278	2015	Turkey	female	59	bacterial infection	blood	7828835
Mer-277	2015	Turkey	female	69	bacterial infection	blood	7828836
Mer-274	2015	Turkey	male	78	bacterial infection	blood	7828842

Mer-271	2015	Turkey	female	74	bacterial infection	blood	7828810
Mer-267	2015	Turkey	female	42	bacterial infection	blood	7828812
Mer-266	2015	Turkey	male	72	bacterial infection	blood	7828811
Mer-262	2015	Turkey	female	85	bacterial infection	blood	7828813
Mer-259	2016	Singapore	male	64	bacterial infection	blood	7828816
Mer-257	2016	Singapore	female	44	bacterial infection	blood	7828643
Mer-254	2016	Singapore	male	72	bacterial infection	blood	7828798
Mer-253	2015	Singapore	female	67	bacterial infection	blood	7828793
Mer-252	2016	Singapore	male	63	bacterial infection	blood	7828794
Mer-251	2016	Singapore	male	82	bacterial infection	blood	7828795
Mer-246	2016	Singapore	male	72	bacterial infection	blood	7828792
Mer-243	2015	Singapore	female	63	bacterial infection	blood	7828768
Mer-242	2015	Singapore	female	73	bacterial infection	blood	7828767
Mer-241	2015	Singapore	male	55	bacterial infection	blood	7828766
Mer-239	2015	Singapore	male	53	bacterial infection	blood	7828772
Mer-237	2015	New Zealand	male	60	bacterial infection	blood	7828770
Mer-232	2015	Australia	male	78	bacterial infection	blood	7828844
Mer-230	2015	Australia	male	66	bacterial infection	blood	7828848
Mer-227	2016	Australia	female	55	bacterial infection	blood	7828845
Mer-216	2015	Singapore	female	56	bacterial infection	blood	7828657
Mer-215	2015	Singapore	male	76	bacterial infection	blood	7828656
Mer-214	2015	Singapore	male	85	bacterial infection	blood	7828646
Mer-211	2015	Singapore	female	67	bacterial infection	blood	7828819
Mer-210	2015	Singapore	female	59	bacterial infection	blood	7828663
Mer-209	2015	Singapore	female	67	bacterial infection	blood	7828662

Mer-207	2015	Singapore	male	92	bacterial infection	blood	7828664
Mer-206	2015	Singapore	male	61	bacterial infection	blood	7828641
Mer-205	2015	Singapore	female	78	bacterial infection	blood	7828642
Mer-202	2015	Singapore	female	61	bacterial infection	blood	7828738
Mer-199	2015	Singapore	male	70	bacterial infection	blood	7828633

Table S2: Metadata of isolates applied for phylogenetic analysis.

Isolates ID	Aminoglycoside	Beta-lactam	Colistin	Fluoroquinolone	Fosfomycin	MLS	Phenicol	Sulphonamide	Tetracycline	Trimethoprim
Mer-304	*aph(3'')-lb, aadA5, aph(6)-ld*	*blaCTX-M-27*		*parC p.S80I, parE p.L445H, gyrA p. S83L, gyrA p. D87N*		*mdf(A)**, *mph(A)*		*sul1, sul2*	*tet(A)*	*dfrA17**
Mer-335	*aadA5*	*blaCTX-M-14*		*parC p.S80I, parC p.E84V, parE p.I529L, gyrA p. S83L, gyrA p. D87N*		*mdf(A)**, *mph(A)*		*sul1*	*tet(B)*	*dfrA17*
Mer-277	*aph(3'')-lb, aph(6)-ld*	*blaCTX-M-15, blaTEM-1B*		*parE p.I529L, gyrA p. S83L*		*mdf(A)**		*sul2*	*tet(A)*	
Mer-310	*aph(3'')-lb, aadA5, aac(3)-lld**, *aph(6)-ld*	*blaCTX-M-27, blaTEM-1B*		*parE p.I529L, gyrA p. S83L*		*mdf(A)**, *mph(A)*		*sul1, sul2*	*tet(A)*	*dfrA17*
Mer-342		*blaCTX-M-27, blaTEM-1B*		*parE p.I529L, gyrA p. S83L*		*mdf(A)**				

Table S3. Resistance gene list of ST131 clade A isolates.

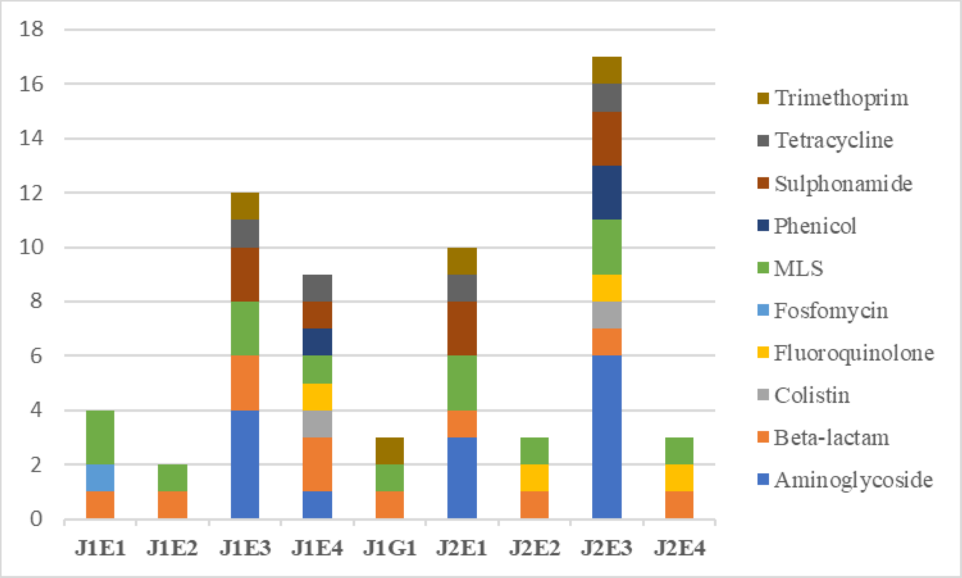

Fig S1. The number of AMR genes subject to different classes detected by ResFinder. The acquired AMR genes were detected with ResFinder as the default setting, only the acquired resistance genes were counted, the mutations are not shown here. The details of AMR profiling are shown in Table 2. MLS: macrolide, lincosamide, and streptogramin B.

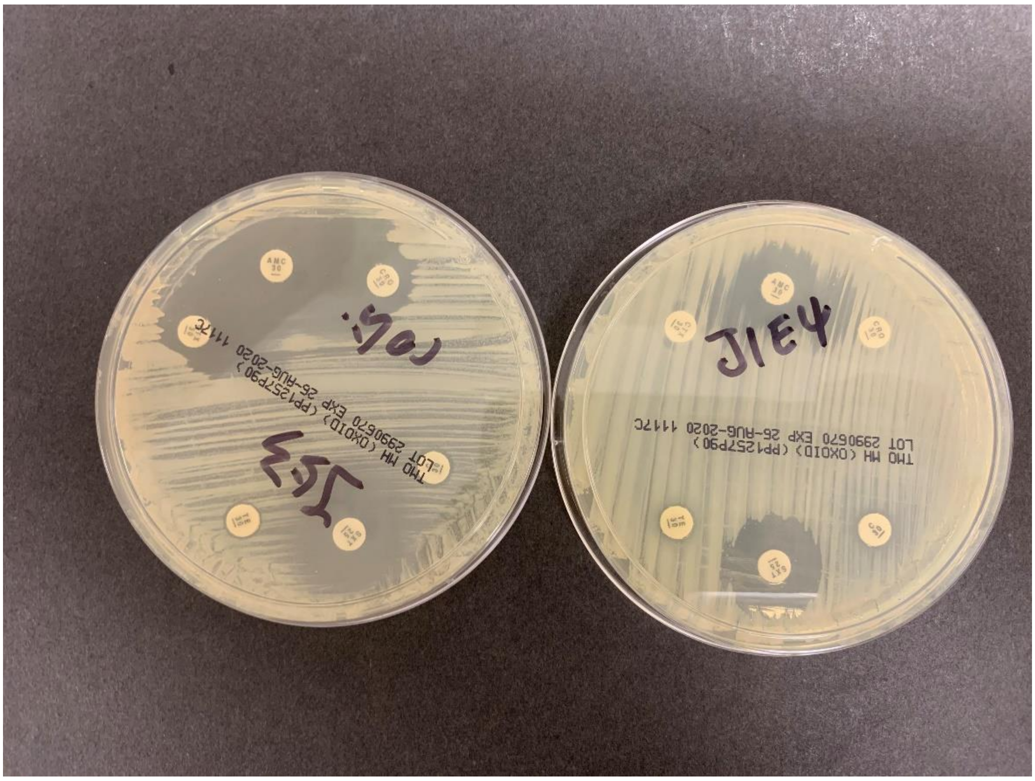

Fig S2. The comparison of Disc diffusion result between the donor J1E4 and the transconjugant J53coli. Not like the donor, the transconjugant is less resistant to cefotaxime and ceftriaxone, without clear ESBL-producing phenotype. But the resistance to tetracycline and chloramphenicol was co-conjugated together with the colistin resistance.

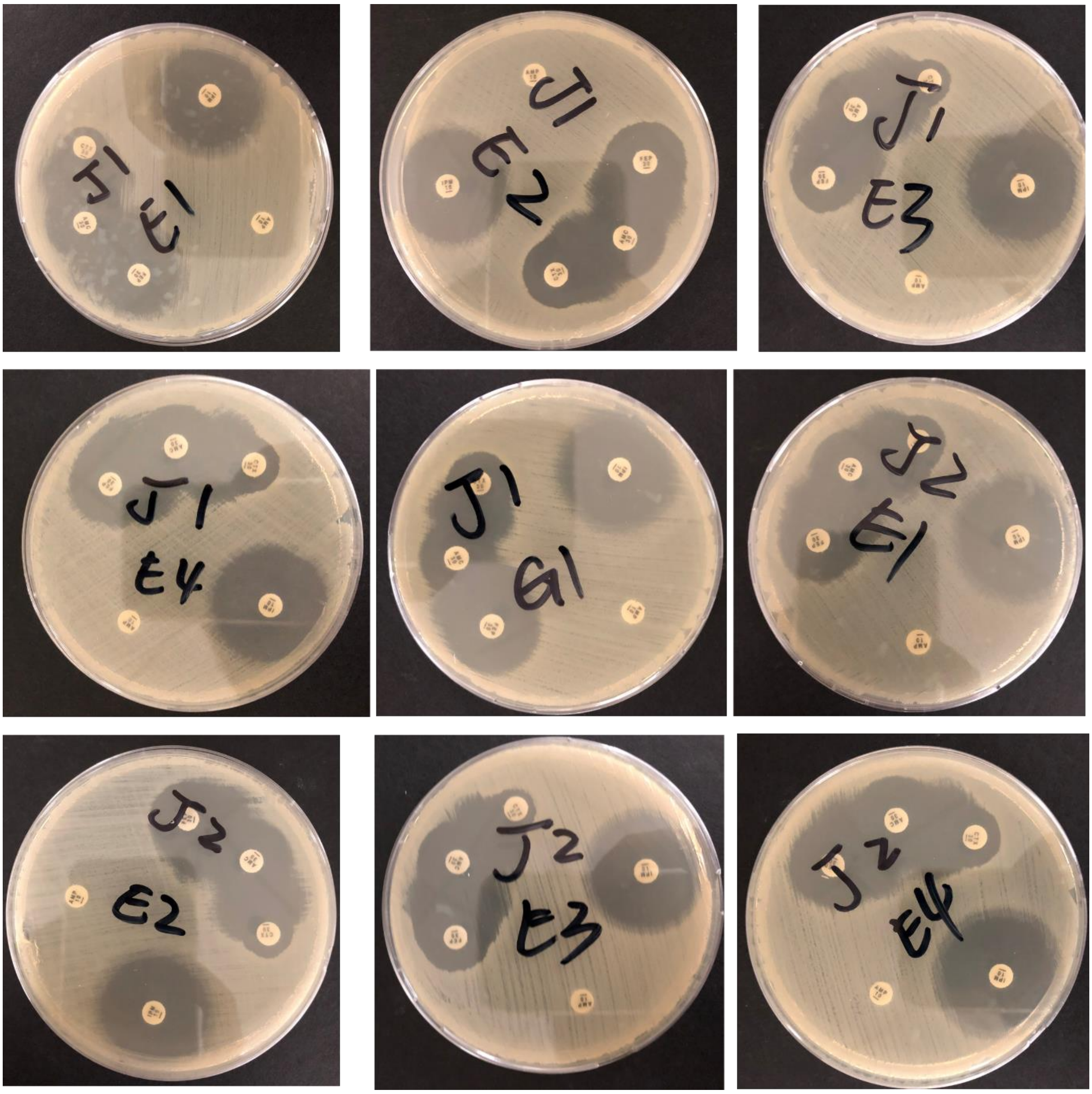

Fig S3. The results of the double-disc synergy test. Discs of amoxicillin/clavulanic acid (AMC, 30 μg) were placed between the cefotaxime (CTX, 30 μg) and cefepime (FEP, 30 μg). The positive results were shown in all sequenced nine isolates as elliptical clearing was apparent between the AMC disc and CTX as well as FEP discs. Imipenem (IMP, 10 μg) and ampicillin (AMP, 10 μg) were also included for comparison.

Isolate ID	Resistance information		Contigs information			BLASTn output for the best hit for the contigs containing resistance genes			
	Related resistance	Resistance genes	Contigs ID	Contigs length/bp	Coverange	Total score	Query cover	E value	Identity
J1E1#	Beta-lactams	*bla*$_{CTX\text{-}M\text{-}15}$	27	269951#	62.7856	3.51E+04	99%	0	98.34%
J1E2	Beta-lactams	*bla*$_{CTX\text{-}M\text{-}8}$	38	2271	80.963	5.01E+03	100%	0	100.00%
J1E3	Beta-lactams	*bla*$_{TEM\text{-}1B}$	33	7641	58.0969	1.60E+04	100%	0	100.00%
	Beta-lactams	*bla*$_{CTX\text{-}M\text{-}15}$	151	128211	62.4876	2.62E+05	97%	0	99.90%
J1E4	Colistin	*mcr-1.1*	3	35325	40.6145	3.33E+04	52%	0	97.08%
	Beta-lactams	*bla*$_{TEM\text{-}1B}$	71	2463	36.5245	7.82E+03	100%	0	100.00%
	fluoroquinolone	*qnrS1*	59	19817	38.2183	4.33E+04	86%	0	99.94%
	Beta-lactams	*bla*$_{CTX\text{-}M\text{-}15}$	28	128728	66.8204	2.31E+05	96%	0	99.61%
J1G1	Beta-lactams	*bla*$_{CTX\text{-}M\text{-}14}$	80	110660	54.303	1.69E+05	83%	0	100.00%
J2E1	Beta-lactams	*bla*$_{CTX\text{-}M\text{-}27}$	72	1651	68.5348	5.32E+03	100%	0	100.00%
J2E2#	Fluoroquinolone	*qnrS1*	31	289403#	56.8063	2.96E+04	80%	0	99.98%
	Beta-lactams	*bla*$_{CTX\text{-}M\text{-}15}$	31	289403#	56.8063				
J2E3	Fluoroquinolone	*qnrS1**	39	15173	43.6608	3.32E+04	100%	0	99.93%
	Beta-lactams	*bla*$_{CTX\text{-}M\text{-}55}$	39	15173	43.6608	3.32E+04	100%	0	99.93%
	Colistin	*mcr-3.1*	72	5509	39.0384	2.36E+04	100%	0	100.00%
J2E4#	Beta-lactams	*bla*$_{CTX\text{-}M\text{-}15}$	220	716717#	69.569	2.11E+04	56%	0	100%
	Fluoroquinolone	*qnrS1*	220	716717#	69.569				

Isolate ID	Best hit genome determined by Blastn					
	Reference ID	Location	Plasmid type	Country	Source	Accession
J1E1#	*E. coli* E-1246	chromosome	N.A.	Sweden	Human	CP025573.1
J1E2	*E. coli* pLV23529-CTX-M-8	plasmid	IncX4	Portugal	animal (swine)	KY964068.1
J1E3	*E. coli* pU14A	plasmid	IncFIB	Australia	human	CP035517.1
	E. coli BH100	chromosome	N.A.	Brazil	human (urine)	CP024650.2
J1E4	*E. coli* pEC2-4	plasmid	IncFIA(HI1)/IncHI1A/IncHI1B(R27)	Malaysia	animal (swine)	CP016184.1
	E. coli p1919D3-1	plasmid	IncFIA(Hl1)*/IncHI1A/IncHI1B(R27)	China	animal (swine)	CP046004.1
	E. coli pEC2-4	plasmid	IncFIA(HI1)/IncHI1A/IncHI1B(R27)	Malaysia	animal (swine)	CP016184.1
	E. coli WI2	chromosome	N.A.	France	human	LT838200.1
J1G1	*E.coli* MS14385	chromosome	N.A.	Australia	human(blood)	LR130555.1
J2E1	*E. coli* p146-1	plasmid	IncFIA*/IncFIB(AP001918)*	USA	human	CP041573.1
J2E2#	*Klebsiella pneumoniae* pR210-2-CTX	plasmid	IncFII(K)*	China	human	CP034085.1
J2E3	*Salmonella* spp. pCFSA1096	plasmid	IncHI2A*	China	food	CP033347.1
	Salmonella spp. pCFSA1096	plasmid	IncHI2A*	China	food	CP033347.2
	E. coli pJSWP006_1	plasmid	IncFIA*/IncFIB(AP001918)*	Japan	enviroment (sewage)	AP018939.2
J2E4#	*Klebsiella pneumoniae* pR210-2-CTX	plasmid	IncFII(K)*	China	human	CP034085.1

Table S4. Best hits of the contigs harboring AMR genes found by Blastn. The hits were selected based on both coverage and identification. #: the size of the contigs was over the blast size limit, a 20000 bp fragment contain the AMR genes were extracted for blast instead. *: identification below 100%

**Antimicrobials (Disc Content)**	**J1E1**	**J1E2**	**J1E3**	**J1E4**	**J2E1**	**J2E2**	**J2E3**	**J2E4**	**J1G1**	**Zone Diameter Breakpoints (mm) (CLSI-2020-M-100-ED30, Table 2A)**
Chloramphenicol (30 µg)	22	24	26	0	23	24	0	21	24	12
Trimethoprim-sulfamethoxazole (25 µg)	10	27	0	19	0	27	0	24	25	10
Tetracycline (30 µg)	21	21	8	0	0	22	0	20	22	11

Table S5: Diameter of the inhibit zone for disc diffusion. Resistant and sensitive were defined based on the breakpoints of CLSI-M100, Red: resistant, green: sensitive.

Primer name	Sequence 5' to 3' direction	Application products size /bp	References
TEM-164.SE	tcgccgcatacactattctcagaatga	445	[1]
TEM-165.AS	acgctcaccggctccagatttat		
CTX-M-U1	atgtgcagyaccagtaargtkatggc	593	[2]
CTX-M-U2	tgggtraartargtsaccagaaycagcgg		
MCR-1-FW	AGTCCGTTTGTTCTTGTGGC	320	[3]
MCR-1-REV	AGATCCTTGGTCTCGGCTTG		

Table S6. Primers used for the PCR test.

1. MONSTEIN, H.J., et al., *Multiplex PCR amplification assay for the detection of blaSHV, blaTEM and blaCTX-M genes in Enterobacteriaceae.* Apmis, 2007. **115**(12): p. 1400-1408.
2. Boyd, D.A., et al., *Complete nucleotide sequence of a 92-kilobase plasmid harboring the CTX-M-15 extended-spectrum beta-lactamase involved in an outbreak in long-term-care facilities in Toronto, Canada.* Antimicrobial agents and chemotherapy, 2004. **48**(10): p. 3758-3764.
3. Rebelo, A.R., et al., *Multiplex PCR for detection of plasmid-mediated colistin resistance determinants, mcr-1, mcr-2, mcr-3, mcr-4 and mcr-5 for surveillance purposes.* Eurosurveillance, 2018. **23**(6): p. 17-00672.
